# Deciphering Metazoan Community Dynamics Using eDNA in a Human-Impacted Gulf Ecosystem: Spatiotemporal Patterns and Environmental Drivers

**DOI:** 10.3390/ani16091322

**Published:** 2026-04-26

**Authors:** Shiyun Fang, Lihong Gan, Tianhao Yao, Hengsong Wu, Wenjian Chen, Yusen Li, Bo Huang, Lei Zhou

**Affiliations:** 1 University Joint Laboratory of Guangdong Province, Hong Kong and Macao Region on Marine Bioresource Conservation and Exploitation, College of Marine Sciences, South China Agricultural University, Guangzhou 510642, China; fangshiyun@stu.scau.edu.cn (S.F.);; 2 Key Laboratory of Aquaculture Genetic and Breeding and Healthy Aquaculture of Guangxi, Guangxi Academy of Fishery Sciences, Nanning 530021, China; 3 Engineering Research Center of Hongshui River Rare Fish Conservation, Guangxi Zhuang Autonomous Region, Guiping 537200, China

**Keywords:** environmental DNA (eDNA) metabarcoding, metazoa, coastal, COI gene, anthropogenic pressures

## Abstract

Coastal ecosystems, particularly semi-enclosed gulfs, are increasing anthropogenic pressures from urbanization and industrialization with profound impacts on biodiversity maintenance, energy transfer, and biogeochemical cycling. However, how metazoan communities—key components of marine food webs—respond to spatiotemporal variability and human disturbance remains insufficiently understood. This study applied eDNA metabarcoding targeting the mitochondrial COI gene to investigate metazoan communities across 68 stations in the Beibu Gulf, spanning bay, coastal, and island regions, during wet and dry seasons. In total, 878 metazoan ASVs from 13 phyla were detected. Arthropoda dominated both seasons (wet: 85%; dry: 55%), whereas Chordata increased during the dry season (wet: 0.16%; dry: 37%). At the α-diversity level, diversity peaked in the bay region during the dry season and shifted toward the coastal region during the wet season. At the β-diversity level, community composition differed significantly between seasons and spatial regions, with seasonal variation exerting a stronger influence than spatial differentiation. Co-occurrence networks revealed higher complexity during the dry season. β-diversity was overwhelmingly driven by species turnover (94.4%). The island region exhibited the highest community uniqueness, while the human-impacted bay region showed reduced distinctiveness. Redundancy analysis further identified anthropogenically influenced inorganic nitrogen, together with water temperature, transparency, and salinity, as key environmental drivers shaping community structure. βNTI analysis indicated that community assembly was governed by the combined effects of deterministic and stochastic processes. Overall, this study highlights how environmental gradients and human pressures jointly regulate metazoan dynamics, providing insights for biodiversity conservation in human-impacted coastal seas.

## Simple Summary

Coastal seas are increasingly affected by human activities such as urban expansion and industrial development, which can disrupt marine life and ecosystem stability. Semi-enclosed gulfs are particularly vulnerable because water exchange is limited, allowing pollutants and nutrients to accumulate more easily. In this study, we explored how small marine animals, which form the base of ocean food webs, change across seasons and under different levels of human influence in the Beibu Gulf. Our results show that seasonal environmental changes play a stronger role than location in shaping these communities. At the same time, areas with intense human activity tend to lose their unique biological characteristics, while less disturbed island regions still maintain distinct communities. These findings highlight the combined influence of natural variability and human pressure on marine ecosystems. Understanding these patterns is important for improving conservation strategies and supporting sustainable management in coastal regions facing rapid development.

## 1. Introduction

Coastal ecosystems, as the interface between land and ocean, are not only among the most biodiverse and productive regions globally [1] but also serve as key areas for maintaining global marine ecological balance and biogeochemical cycles [2,3]. However, with the intensification of global urbanization and industrialization, coastal waters are facing unprecedented anthropogenic pressures [4]. Existing studies have shown that terrestrial nutrient inputs [5,6], industrial wastewater discharge [7], shipping activities, and overfishing [8,9] are profoundly altering the physicochemical environment of coastal waters. These cumulative effects not only lead to water eutrophication and pollutant accumulation but also change the structure of biological communities [10,11,12].

As a core component of the marine food web, Metazoa encompass multiple phyla, ranging from microbenthic organisms to large invertebrates, acting as an energy bridge between primary producers and higher trophic levels. Due to their high sensitivity to environmental changes, metazoan community succession has become an important biological indicator for monitoring the health of coastal ecosystems [13]. Despite their critical ecological roles, traditional morphology-based surveys suffer from inherent limitations in detecting micro-larvae, early life stages, and cryptic taxa, and are often labor-intensive and taxonomically biased [14,15,16]. These constraints hinder comprehensive characterization of spatiotemporal community dynamics in structurally complex habitats, highlighting an urgent need for more efficient and precise biodiversity assessments in human-impacted, sensitive coastal areas.

In contrast, environmental DNA (eDNA) approaches enable non-invasive, high-throughput, and taxonomically inclusive biodiversity detection, capturing rare and elusive species across multiple life stages. Owing to their high sensitivity, scalability, and reproducibility, eDNA metabarcoding has emerged as an effective tool for monitoring changes in marine ecosystem structure and dynamics [17,18]. Among the molecular markers available for metabarcoding, the mitochondrial cytochrome c oxidase subunit I (COI) gene is the most widely used DNA barcode for animals. Owing to its moderate evolutionary rate and characteristic alternation of conserved and highly variable regions, COI supports both universal primer design and robust species-level discrimination. In metazoan-focused studies, COI metabarcoding enables robust taxonomic assignment across multiple phyla, including Arthropoda, Mollusca, Annelida, and Chordata. Compared with more conserved markers such as 18S rRNA, the COI gene provides higher taxonomic resolution and captures subtle interspecific variation, thereby offering distinct advantages for resolving community responses to environmental gradients and pollution-related disturbances [19,20,21,22].

eDNA-based metabarcoding has been widely applied in biodiversity assessments of coastal waters, demonstrating strong potential for characterizing community composition and supporting ecological monitoring. To date, most applications have focused on microbial assemblages and fishes, substantially advancing our understanding of coastal ecosystem structure, dynamics, and functioning [23,24,25,26]. However, the spatiotemporal responses of metazoan communities to interacting seasonal variability and anthropogenic disturbances have yet to be comprehensively evaluated, representing a critical gap in understanding coastal ecosystem dynamics.

The Beibu Gulf, located in the northwestern South China Sea, is a typical semi-enclosed basin characterized by seasonally dynamic and complex environmental conditions, supporting abundant fishery resources [27]. In recent decades, rapid socio-economic development associated with the Beibu Gulf Economic Zone has intensified anthropogenic pressures, making the region a representative natural laboratory for examining coastal ecosystem responses to human disturbances. Such pressure is manifested in trace-element enrichment in atmospheric deposition associated with industrial activities [28], rising sediment accumulation rates in the northern Beibu Gulf linked to intensified coastal development [29], and elevated nutrient concentrations accompanied by eutrophication in some nearshore bays [30]. Moreover, previous studies have demonstrated that riverine inputs and effluents from coastal aquaculture have altered nutrient regimes in nearshore waters, leading to pronounced shifts in biological community composition [30,31]. Against this background, elucidating spatiotemporal patterns of metazoan community dynamics in the Beibu Gulf is essential for understanding ecosystem responses to cumulative anthropogenic stressors and for informing the sustainable management of key nearshore ecological functional zones in China.

In this study, we applied eDNA metabarcoding targeting the mitochondrial COI gene to characterize the spatiotemporal patterns of metazoan α- and β-diversity in the Beibu Gulf and to examine how environmental gradients and anthropogenic pressures drive these patterns. By integrating diversity metrics, community composition, and environmental variables, this study provides a systematic assessment of metazoan community heterogeneity in a human-impacted semi-enclosed coastal system, offering insights into the ecological processes shaping biodiversity patterns in the Beibu Gulf.

## 2. Materials and Methods

### 2.1. Study Area and Sample Collection

**Figure 1:**
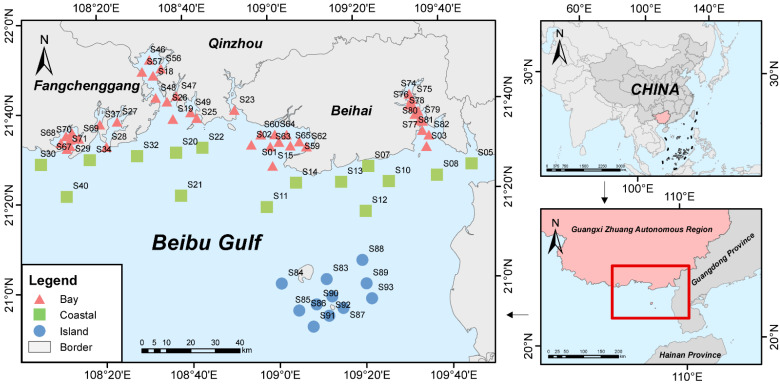
Sampling sites and study area of the Beibu Gulf.

In this study, a total of 68 sampling stations were established across the Beibu Gulf region. Water samples were collected from all stations during two seasons: the wet season (October 2022) and the dry season (January 2023). In terms of spatial distribution, the stations were divided into three zones: bay, coastal, and island region (Figure 1).

At each sampling station, three surface seawater samples were collected at a depth of approximately 0.5 m using an acrylic water sampler. Filtration was conducted immediately on site with a sterile glass vacuum filtration unit fitted with autoclaved 0.22 μm polycarbonate membranes (Millipore, Burlington, MA, USA) until the filter became visibly clogged, with approximately 1 L of seawater filtered per sample. Following filtration, membranes were placed in sterile 50 mL centrifuge tubes and stored at −80 °C until DNA extraction. To minimize contamination, bottles and filtration equipment were soaked in 10% sodium hypochlorite and thoroughly rinsed with sterile water before each sampling event. All sampling bottles were pre-rinsed with the sampling water before use. Sterile water was included as a negative control to verify the absence of contamination.

### 2.2. Measurement of Environmental Factors

Field-based parameters, including water temperature, pH, salinity, and dissolved oxygen (DO), were recorded in situ using a YSI ProPlus (YSI, Yellow Springs, OH, USA) multiparameter probe, with transparency determined by a Secchi disk. For laboratory-based chemical analysis, Chlorophyll-a (Chl-a) was quantified via the extraction fluorometric method. A suite of spectrophotometric and colorimetric techniques was employed to determine nutrient concentrations, specifically for NH_4_^+^-N, NO_3_^−^-N, NO_2_^−^-N, total phosphorus (TP), active phosphate, and active silicate. Additionally, suspended solids (SSs) were analyzed gravimetrically, while an Elementar Vario EL III analyzer was utilized to measure elemental compositions, including total nitrogen (TN), total organic carbon (TOC), and dissolved organic carbon (DOC).

### 2.3. DNA Extraction and High-Throughput Sequencing

Total DNA was extracted from filter membranes using the DNeasy PowerWater Kit (QIAGEN, Germantown, MD, USA) following the manufacturer’s instructions. DNA purity and concentration were assessed using a NanoDrop One spectrophotometer (Thermo Fisher Scientific, Wilmington, DE, USA) and a Qubit 4.0 Fluorometer (Thermo Fisher Scientific), respectively, retaining samples with OD260/OD280 ratios of 1.8–2.0 and DNA concentrations of 20–100 ng/µL. A fragment of the mitochondrial COI gene was amplified in triplicate using primers m1COIintF and LoboR1 with Phusion High-Fidelity PCR Master Mix. Amplicons were purified, pooled, and used for library construction. Sequencing was conducted on an Illumina NovaSeq 6000 platform (Illumina, San Diego, CA, USA) with a 250 bp paired-end sequencing strategy. Raw reads were quality-filtered and adapter-trimmed using fastp v0.23.2. Denoising and amplicon sequence variant (ASV) inference were performed using a denoising pipeline based on UNOISE3 implemented in USEARCH v10.0, generating an ASV count table. Representative ASV sequences were aligned for downstream analyses, and taxonomic assignment was conducted using the SINTAX algorithm against the MIDORI2 database. ASVs assigned to non-target taxa (e.g., protists, fungi, algae, and terrestrial mammals) were removed prior to downstream analyses. After filtering, 878 ASVs were retained for subsequent community analyses.

### 2.4. Statistical Analyses

All statistical analyses were performed in R v4.3.3, and figures were generated using the ggplot2 package (v4.0.3). The relative read abundance (RRA) of taxa was calculated at the phylum level, and stacked bar plots were constructed to visualize community composition. Correlations among ASVs were calculated based on Spearman’s correlation coefficients, and their significance was tested using the psych package (v2.6.3) (|ρ| ≥ 0.5, *p* < 0.01). Network analyses were conducted using the igraph package (v2.3.0). The modularity index was calculated using the Louvain community detection algorithm, and nodes were classified into topological roles within the network according to their within-module degree (Z) and participation coefficient (P). α-diversity was evaluated using the Shannon diversity index and the evenness index, both calculated with the vegan package (v2.7-3). Differences in α-diversity were assessed using the Kruskal–Wallis test and the Wilcoxon rank-sum test. Differential taxa among groups were identified using the LEfSe method (LDA > 2.0). β-diversity was calculated based on Bray–Curtis and Jaccard distances, and principal coordinates analysis (PCoA) was applied to visualize community dissimilarities. Differences among groups were tested using permutational multivariate analysis of variance (PERMANOVA; adonis2 function in vegan). The Mantel test was used to assess the relationship between community similarity and geographic distance, thereby evaluating the distance–decay pattern. Community specificity was quantified using local contributions to beta diversity (LCBD) and species contributions to beta diversity (SCBD) [32]. LCBD represents the degree of community uniqueness at a particular sampling site compared to the regional average, with the sum of all individual LCBD values in the dataset equaling 1. SCBD, conversely, identifies the specific taxa that contribute most to the overall variation in community composition. β-diversity was further partitioned into turnover and nestedness components. Community assembly processes were assessed using the phylogeny-based metric β nearest taxon index (βNTI). βNTI was calculated based on null model randomizations to quantify the standardized deviation between observed and expected phylogenetic turnover. A threshold of |βNTI| > 2 was used to indicate deterministic processes, with βNTI > 2 representing variable selection and βNTI < −2 indicating homogeneous selection, whereas |βNTI| ≤ 2 suggests that stochastic processes dominate community assembly. The relative contributions of different processes were quantified as the proportion of pairwise comparisons assigned to each category. Differences among seasons and spatial groups were compared using the picante package (v1.8.2). Environmental drivers were analyzed using redundancy analysis (RDA). The significance of explanatory variables was assessed by Monte Carlo permutation tests with 999 iterations. Only significant variables with *p* < 0.05 were retained for the RDA biplot. Non-metric multidimensional scaling (NMDS) and generalized additive model (GAM) contours were used to visualize community gradients. To avoid information redundancy caused by high multicollinearity, environmental factors with lower inter-correlation were prioritized for GAM analysis. Specifically, inorganic N (Inorg–N) was selected to represent the primary nitrogen gradient due to its peak explanatory power, while NH_4_^+^-N was included as a relatively independent nitrogen source with Pearson correlation coefficients below 0.58 against other nitrogen species. Statistical significance was set at *p* < 0.05.

## 3. Results

**Figure 2:**
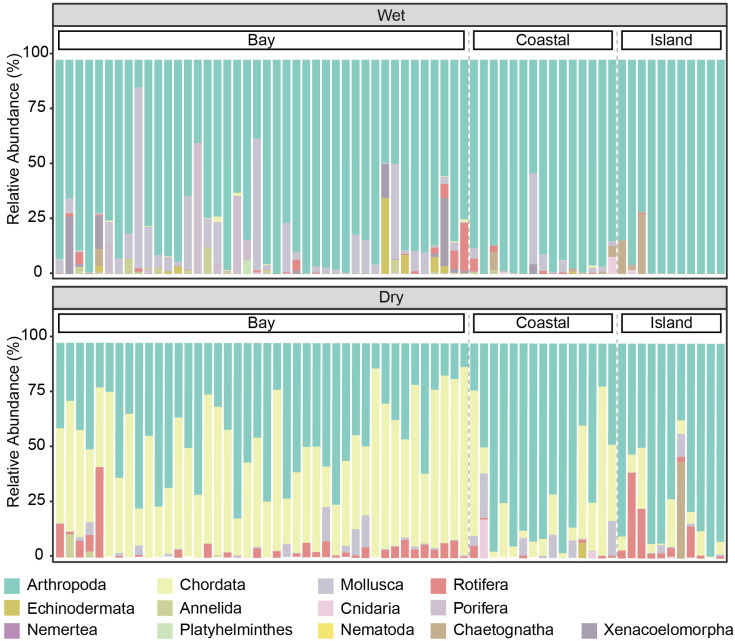
Community composition of metazoan communities during wet and dry seasons.

A total of 878 metazoan ASVs were identified, belonging to 13 phyla, 24 classes, 57 orders, 76 families, 102 genera, and 119 species. At the phylum level, only a few phyla accounted for most of the relative read abundance (Figure 2). Arthropoda showed clear predominance across all samples, accounting for 85% of the total sequence reads in the wet season and 55% in the dry season. Chordata ranked second, with relative read abundance below 1% in the wet season but increasing to 37% in the dry season (Figure S1).

**Figure 3:**
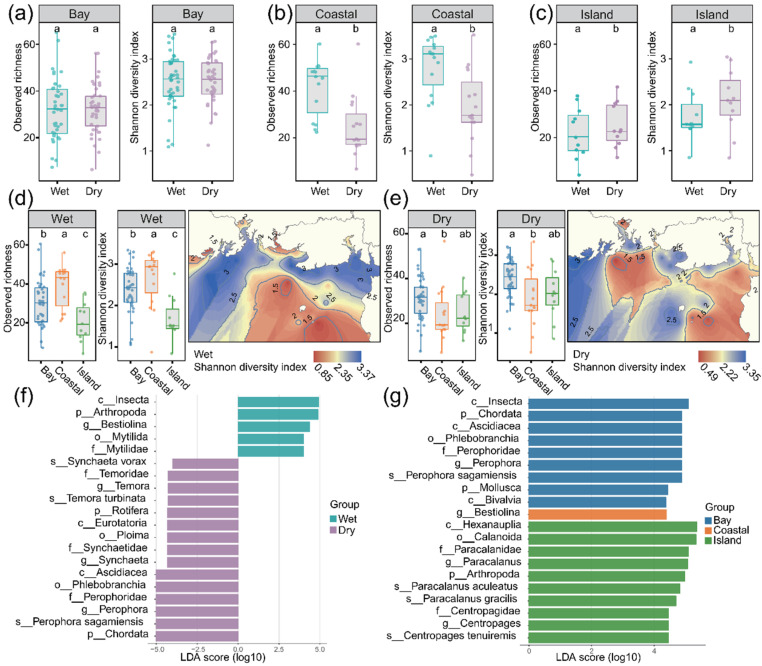
Spatiotemporal variation in alpha-diversity and key differential taxa. Seasonal differences in observed species richness and Shannon diversity in the bay (**a**), coastal region (**b**), and island region (**c**). Spatial differences in Shannon diversity among the bay, coastal region, and island region during the wet (**d**) and dry (**e**) seasons. LEfSe-identified biomarkers showing significant differences between seasons (**f**). LEfSe-identified biomarkers showing significant spatial differences among the bay, coastal region, and island region (**g**) (p: phylum, c: class, o: order, f: family, g: genus, s: species). Different letters above the boxes indicate statistically significant differences among groups at *p* < 0.05, determined by the Kruskal–Wallis test followed by Dunn’s post hoc multiple comparisons test.

Observed species richness and Shannon index exhibited highly spatiotemporal patterns. Seasonally, significant differences were observed in coastal and island regions, with higher diversity in the wet season for coastal samples and in the dry season for island samples, whereas the bay samples remained relatively stable (Figure 3a–c). Spatially, during the wet season, the coastal region showed the highest richness and diversity, significantly exceeding those of both the bay and island regions (Figure 3d). In the dry season, indices in the bay were significantly higher than those in the coastal region, while the island region exhibited intermediate values with no significant difference from either the bay or coastal region (Figure 3e). ACE richness showed patterns largely consistent with those of observed richness and Shannon diversity, except in the island region where values were higher in the wet season than in the dry season (Figure S2). This overall consistency further supports the robustness of the observed spatiotemporal patterns.

To pinpoint taxa responsible for the observed compositional shifts, LEfSe analysis was applied to identify differentially abundant biomarkers. Numerous taxa differed significantly between seasons (Figure 3f). The wet season was enriched in arthropod lineages, particularly insect-related taxa and Bestiolina, together with elevated signals from bivalve-associated groups. In contrast, the dry season showed higher relative read abundances of chordate and ascidian lineages, accompanied by rotifers and calanoid copepods such as Synchaeta and Temora. Spatially, LEfSe identified distinct sets of biomarkers among habitats (Figure 3g). The bay region was mainly characterized by insect-, ascidian-, and bivalve-related taxa, whereas the island region was enriched in calanoid copepod lineages, especially taxa related to Paracalanus and Centropages. The coastal region displayed a more restricted biomarker profile, with Bestiolina emerging as the dominant indicator taxon.

**Figure 4:**
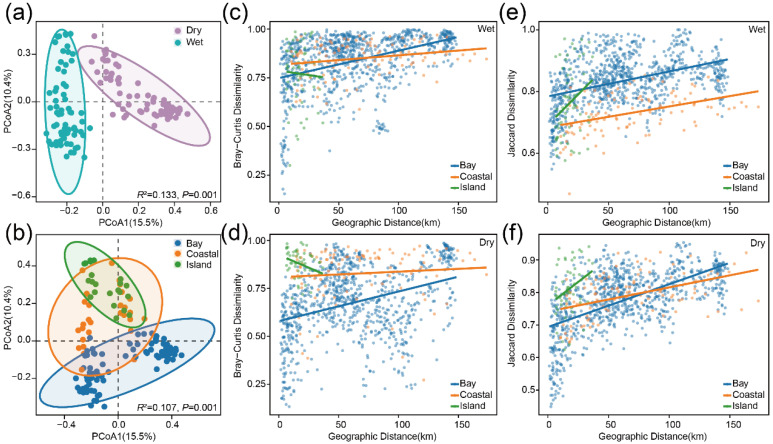
β-diversity patterns and distance–decay relationships of metazoan communities. Temporal (**a**) and spatial (**b**) variation in community composition. Distance–decay relationships based on the Bray–Curtis index during the wet (**c**) and dry (**d**) seasons. Distance–decay relationships based on the Jaccard index during the wet (**e**) and dry (**f**) seasons.

Based on the Bray–Curtis index, PCoA was used to visualize differences in metazoan community composition among groups, and PERMANOVA was applied to statistically test intergroup differences. PCoA1 and PCoA2 together explained 25.9% of the total variation. Temporally, there was a significant difference in community composition between the wet and dry seasons (*p* = 0.001), indicating a pronounced compositional divergence between the two communities (Figure 4a). Spatially, significant differences were also detected among the bay, coastal, and island samples (*p* = 0.001), with coastal and island samples showing more similar community compositions (Figure 4b). Seasonal differences explained a slightly larger proportion of the variation (R^2^ = 0.133) than spatial differences did (R^2^ = 0.107). Distance–decay patterns varied markedly among spatial settings (Figure 4). The bay exhibited the strongest and most consistent spatial structuring, with clear distance–decay relationships. In the coastal region, distance–decay effects were evident for species composition based on the Jaccard index, whereas RRA-based patterns derived from the Bray–Curtis index were weak or absent. In contrast, island habitats showed little to no distance–decay, and island communities displayed divergent patterns between Bray–Curtis and Jaccard indices (Table S1).

**Figure 5:**
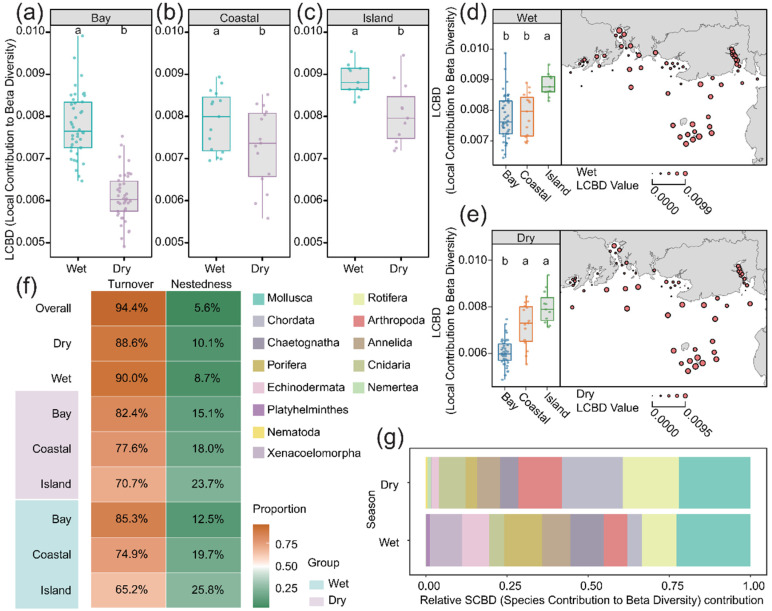
Comparison of LCBD values between wet and dry seasons in the bay (**a**), coastal region (**b**), and island region (**c**). Spatial differences in LCBD among the bay, coastal region, and island region during the wet (**d**) and dry (**e**) seasons. Partitioning of β-diversity into species turnover and nestedness components (**f**). SCBD of major phyla, with Mollusca contributing most to spatial heterogeneity (**g**). Different letters above the boxes indicate statistically significant differences among groups at *p* < 0.05, determined by the Kruskal–Wallis test followed by Dunn’s post hoc multiple comparisons test.

To further characterize the observed β-diversity patterns, β-diversity was partitioned into turnover and nestedness components. Species turnover emerged as the dominant driver of β-diversity, accounting for 94.4% of the total variation, whereas nestedness contributed only 5.6% (Figure 5f). LCBD analysis revealed significant spatiotemporal shifts in community uniqueness, with higher values recorded in the wet season (Figure 5a–c). Spatially, island samples consistently exhibited the highest LCBD values across both seasons, indicating greater community uniqueness, while bay samples showed the lowest values (Figure 5d,e). Additionally, SCBD results identified Mollusca as the primary contributor to community specificity in both seasons (Figure 5g).

**Figure 6:**
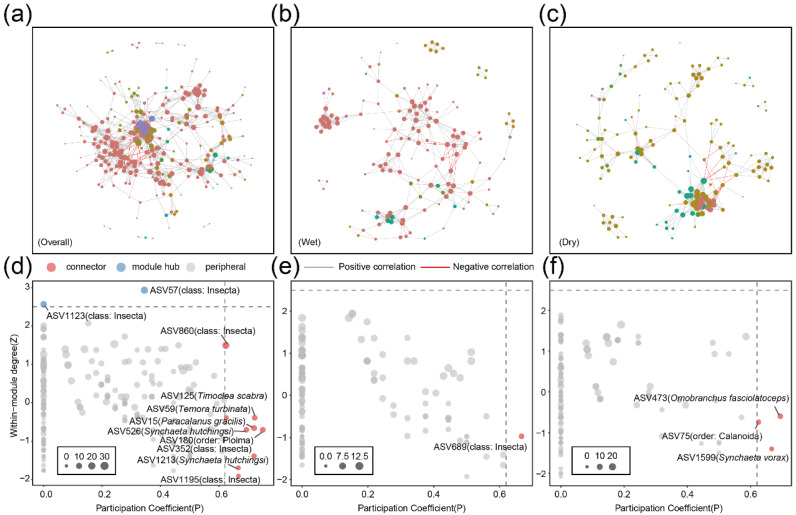
Network structure and topological roles of metazoan communities across seasons. (**a**) All samples combined, (**b**) wet season, and (**c**) dry season. (**d**) Classification of ASVs based on their within-module degree (Z) and participation coefficient (P) in the network of all samples. (**e**) Topological roles of ASVs during the wet season. (**f**) Topological roles of ASVs during the dry season. Each node represents an individual ASV, and edges represent significant Spearman correlations with |ρ| ≥ 0.5 and *p* < 0.01. Colors indicate different phyla and node sizes represent the degree. The threshold values of Z = 2.5 and *p* = 0.62 separate nodes into four categories: peripherals (Z < 2.5, *p* < 0.62), connectors (Z < 2.5, *p* > 0.62), module hubs (Z > 2.5, *p* < 0.62), and network hubs (Z > 2.5, *p* > 0.62).

The network diagrams illustrated co-occurrence patterns among metazoan communities. The number of nodes was similar between the wet and dry seasons, at 147 and 150 respectively. The dry season network exhibited higher complexity, with 514 edges and an average degree of 6.853, compared to 433 edges and an average degree of 5.891 in the wet season. Regarding stability, the natural connectivity of the dry season was 13.3238, significantly higher than the 5.7356 recorded for the wet season. The maximum vulnerability was higher in the wet season at 0.1271, compared to 0.1014 in the dry season. Additionally, positive links predominated in all groups, exceeding 97% in all cases (Table 1, Figure 6b,c). In the overall network, ASV1123 (class: Insecta) and ASV57 (class: Insecta) were identified as module hubs. Additionally, multiple connectors were identified, including ASV860 (class: Insecta), ASV125 (*Timoclea scabra*), ASV59 (*Temora turbinata*), ASV15 (*Paracalanus gracilis*), ASV526 (*Synchaeta hutchingsi*), ASV180 (order: Ploima), ASV352 (class: Insecta), ASV1213 (*Synchaeta hutchingsi*), and ASV1195 (class: Insecta) (Figure 6d). In the wet season network, there was one connector, ASV689 (class: Insecta) (Figure 6e). The dry season network showed three connectors, which were ASV473 (*Omobranchus fasciolatoceps*), ASV75 (order: Calanoida), and ASV1599 (*Synchaeta vorax*) (Figure 6f).

**Figure 7:**
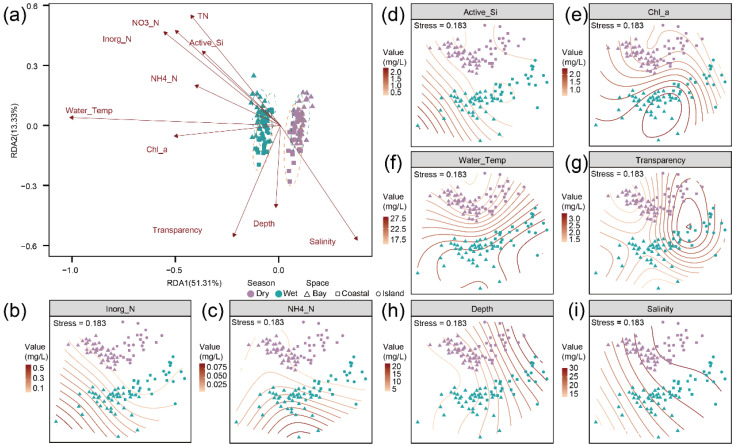
Relationships between metazoan community composition and environmental variables. (**a**) Influence of significant environmental factors on community structure based on RDA. Community distribution along environmental gradients of Inorg-N (**b**), NH_4_^+^–N (**c**), Active-Si (**d**), Chl-a (**e**), water temperature (**f**), transparency (**g**), depth (**h**), and salinity (**i**).

To further explore the environmental drivers underlying the observed community patterns, RDA was performed to examine the relationships between metazoan community composition and environmental factors. The first and second axes explained 51.31% and 13.33% of the total variation, respectively (Figure 7a). Environmental factors including NO_3_^−^-N, TN, Inorg-N, NH_4_^+^-N, Active-Si, Water-Temp, Chl-a, depth, transparency and salinity were significantly correlated with community distribution (*p* < 0.05). NMDS ordination combined with GAM contour plots further indicated that community composition followed clear gradients along several key environmental factors (Figure 7b–i). Correlation analysis showed that most environmental factors were significantly correlated with at least one phylum (Figure S3). Specifically, nutrient concentrations were positively correlated with Porifera, Annelida, Echinodermata, Xenacoelomorpha, Mollusca, and Arthropoda, whereas Chordata and Rotifera exhibited negative correlations with nutrient concentrations. Additionally, Chordata and Arthropoda showed significant associations with water temperature and transparency.

**Figure 8:**
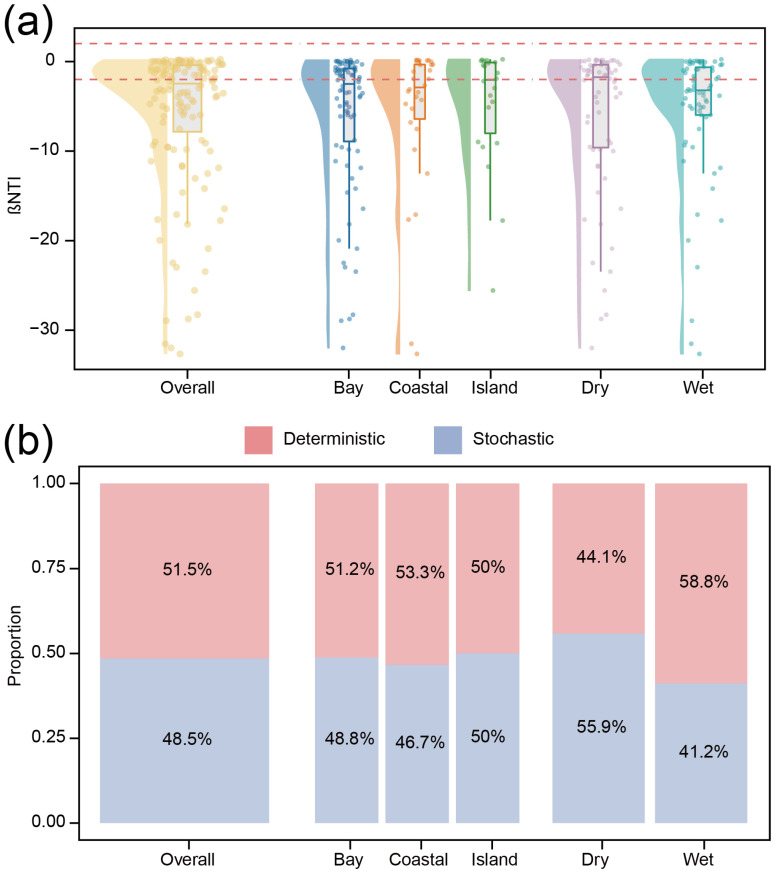
Community assembly processes of metazoan communities. (**a**) Distribution of βNTI values; the red dashed lines represent the thresholds of βNTI at ±2. Values of βNTI > 2 indicate variable selection, βNTI < −2 indicate homogeneous selection, and values between −2 and 2 suggest that stochastic processes dominate community assembly. (**b**) Relative contributions of deterministic and stochastic assembly processes across seasons and spatial groups.

Finally, βNTI analysis revealed that the community assembly was influenced by both deterministic and stochastic processes (Figure 8). The overall distribution of βNTI values was centered around −2, indicating the coexistence of stochastic- and deterministic-influenced assembly processes. When partitioned by season and spatial group, the relative contributions of deterministic and stochastic processes exhibited more pronounced variation between seasons than among spatial groups, while spatial differences remained comparatively small. In the dry season, stochastic processes slightly exceeded deterministic ones (55.9% vs. 44.1%), whereas in the wet season, deterministic processes dominated over stochastic processes (58.8% vs. 41.2%).

## 4. Discussion

### 4.1. Seasonal Reorganization of Metazoan Communities Under Contrasting Hydrological Conditions

Our results demonstrate that seasonal variation represents the primary axis structuring metazoan communities in the Beibu Gulf, exceeding the magnitude of spatial differentiation. Clear seasonal shifts were observed in taxonomic composition, network topology, and community assembly processes, indicating substantial community reorganization rather than simple fluctuations in taxon representation. During the wet season, metazoan communities were overwhelmingly dominated by Arthropoda, accounting for up to 85% of total sequence reads, whereas Chordata increased markedly during the dry season. This pattern is broadly consistent with previous morphology-based observations from Qinzhou Bay, where copepods, a major arthropod group, were identified as the most diverse and one of the most abundant components of the zooplankton community [33]. Such contrasting dominance patterns reflect the strong influence of monsoon-driven hydrological forcing on coastal ecosystems, as previously reported in subtropical and monsoonal marine systems [34,35]. Increased precipitation and runoff during the wet season enhance nutrient loading, water exchange, and primary productivity, creating favorable bottom-up conditions for fast-reproducing and dispersal-efficient arthropod taxa and other opportunistic taxa [36]. In contrast, the relatively stable hydrological regime during the dry season appears more suitable for chordate taxa, which may benefit from clearer waters, reduced turbulence, and altered prey availability. Correlation analyses further confirmed that water temperature was positively associated with Arthropoda and negatively with Chordata.

Co-occurrence network analysis indicates that the metazoan community in the dry season exhibits higher connectivity and a more complex structure than in the wet season, suggesting seasonal shifts in community association patterns. This pattern may be associated with more stable hydrological conditions, reduced freshwater runoff, and weaker environmental variability in the dry season [37], which can enhance niche overlap and stabilize ecological associations. Such stability may promote persistent co-occurrence patterns driven by shared habitat preferences and trophic or indirect interactions [38]. In addition, reduced hydrological connectivity may increase spatial aggregation of organisms, enhancing co-occurrence independent of direct interactions [39]. In contrast, strong hydrological disturbance and nutrient inputs during the wet season [40,41] may disrupt community structure and weaken persistent associations, resulting in a less connected network [42]. Overall, seasonal differences in network structure likely reflect combined effects of environmental stability, spatial processes, and hydrological disturbance. LEfSe analysis further identified Insecta and Rotifera as key seasonal biomarkers. The enrichment of insect-related taxa during the wet season likely reflects enhanced freshwater and brackish inputs transported by runoff, consistent with the sensitivity of these taxa to hydrological connectivity [43,44]. Rotifera, as short-lived and highly responsive planktonic organisms, exhibited rapid responses to seasonal nutrient enrichment and eutrophic conditions, reinforcing their role as indicators of short-term environmental change [45,46,47].

### 4.2. Spatial Heterogeneity, Community Uniqueness, and Conservation Implications

Spatial variation in community composition reflected environmental gradients and anthropogenic influences [48,49]. Bay sites, characterized by high nutrient enrichment and intense anthropogenic disturbance [50], harbored homogenized communities with low LCBD values. This pattern indicates a loss of ecological uniqueness, likely due to strong environmental filtering that favors a similar set of opportunistic or tolerant taxa [51,52]. In contrast, island communities had the highest LCBD values, highlighting their distinct compositions and potential conservation value. Such uniqueness may be related to stronger hydrological isolation among islands, which can constrain dispersal and promote community differentiation [53]. Greater habitat heterogeneity may also contribute to this pattern by shaping local community composition and reinforcing compositional distinctiveness [54]. This interpretation is consistent with evidence that community uniqueness can be associated with both connectivity and habitat heterogeneity [55].

SCBD analyses revealed that Mollusca contributed most to spatial differentiation, followed by Rotifera, Chordata, and Arthropoda. Mollusks, as benthic or sessile organisms, are sensitive to nutrient levels, sediment type, and oxygen availability [56,57], which explains their disproportionate contribution to β-diversity and seasonal variability. Mollusks play a pivotal ecological role in coastal ecosystems by mediating benthic–pelagic coupling through filter feeding, bioturbation, and nutrient recycling [58]. By regulating phytoplankton biomass and facilitating the transformation and redistribution of organic matter [59], mollusks exert strong bottom-up controls on community structure and energy flow. Consequently, spatial and seasonal shifts in molluscan assemblages can amplify community-level differentiation and serve as integrative indicators of environmental change. Their dominant contribution to SCBD therefore highlights not only their responsiveness to environmental gradients but also their ecological importance in structuring metazoan communities in the Beibu Gulf.

β-diversity partitioning revealed that species turnover accounted for 94.4% of total community dissimilarity, whereas nestedness contributed only 5.6%. This indicates that differentiations in metazoan communities were primarily driven by species replacement rather than ordered species loss, a pattern consistent with other marine and freshwater ecosystems experiencing strong environmental gradients [60,61]. Spatially, turnover was highest in bay sites and lowest in island sites, reflecting environmental heterogeneity and anthropogenic influence in the bay versus the unique but internally stable island communities [62]. However, despite lower turnover, island communities exhibited the highest LCBD values, underscoring their role as unique contributors to regional diversity. This decoupling between local uniqueness (LCBD) and within-region turnover underscores the importance of both regional distinctiveness and local environmental variability for sustaining β-diversity [63].

### 4.3. Environmental Drivers and Assembly Processes of Metazoan Communities

βNTI analysis indicated that metazoan community assembly was governed by the combined influence of deterministic and stochastic processes. Spatially, deterministic and stochastic contributions were approximately balanced across regions, each accounting for roughly half of the assembly processes. In contrast, seasonal differences were more pronounced: stochastic processes were slightly more prevalent than deterministic ones during the dry season (55.9% vs. 44.1%), whereas deterministic processes were slightly more prevalent than stochastic processes during the wet season (58.8% vs. 41.2%). Distance–decay patterns further confirmed that community similarity declined with increasing geographic distance, consistent with the combined effects of niche-based and neutral processes [64,65,66]. These patterns suggest that seasonal hydrological forcing modulates the relative importance of environmental filtering and stochastic dispersal. Enhanced runoff and nutrient inputs during the wet season likely strengthen deterministic processes by imposing strong environmental gradients, whereas reduced hydrological connectivity during the dry season may increase the relative role of stochasticity.

Nutrient concentrations (NH_4_^+^–N, Inorg–N, and Active-Si) exhibited strong spatiotemporal gradients, with higher values during the wet season and in nearshore waters. A similar nearshore enrichment pattern has been reported in Tieshan Bay, where DIN, DIP, and COD were negatively correlated with salinity, indicating strong influences of freshwater input and land-based sources [67]. Such a pattern is also consistent with enhanced riverine nutrient loading, which is known to increase nutrient delivery to estuaries and alter nutrient structure and phytoplankton communities in Chinese coastal systems [68]. In aquaculture-influenced coastal waters, untreated pond effluents can further elevate nearshore DIN, phosphate, and Chl-a concentrations, with nutrient levels generally declining offshore [69]. These nutrients likely influence community structure through bottom-up pathways by stimulating primary production, as indicated by elevated Chl-a concentrations in nearshore waters during the wet season. More broadly, anthropogenic nutrient inputs from agriculture, sewage, and aquaculture are widely recognized as important drivers of nutrient enrichment in coastal waters, although their ecological effects are often modulated by local hydrodynamic conditions [70]. Salinity, water temperature, and transparency further modulated community composition by shaping physiological tolerance ranges and habitat suitability. Seasonal increases in suspended particulate matter during the wet season and salinity changes during the dry season likely reflect the combined influence of hydrological forcing and human-derived coastal inputs, reinforcing species replacement along environmental gradients [71,72,73].

## 5. Conclusions

Together, our results demonstrate that metazoan communities in the Beibu Gulf are jointly structured by seasonal forcing, spatial heterogeneity, and environmental filtering, with species turnover emerging as the dominant driver of β-diversity. The pronounced regional uniqueness of island communities underscores their high conservation value, whereas bay systems exhibit stronger influences of anthropogenic disturbance, highlighting the need for targeted management to alleviate external pressures. Seasonal nutrient inputs and hydrological variability act as key regulators of community reorganization, reinforcing the importance of integrated spatiotemporal monitoring frameworks for effective ecosystem assessment. Looking forward, the incorporation of additional molecular markers and long-term observations will further enhance taxonomic resolution and improve the detection of community responses to environmental change. This study is based on two seasonal sampling events, which may limit the detection of fine-scale community succession. Future studies with higher sampling frequency or multi-year monitoring are recommended to better capture temporal dynamics and improve ecological interpretation. Collectively, these findings provide a robust ecological basis for biodiversity conservation and management in coastal marine systems and offer broader insights into the dynamics of semi-enclosed gulfs under intensifying human disturbance and global change.

## Figures and Tables

**Table 1:** Summary of topological properties for the wet, dry, and overall metazoan co-occurrence networks.

Network	Wet	Dry	Overall
Nodes	147	150	237
Edges	433	514	971
Positive Proportion	0.9723	0.9747	0.9743
Average Degree	5.891	6.853	8.194
Natural Connectivity	5.7356	13.3238	13.3304
Maximum Vulnerability	0.1271	0.1014	0.0293

## Data Availability

The data presented in this study are available on request from the authors.

## Supplementary Materials

The following supporting information can be downloaded at https://www.mdpi.com/article/10.3390/ani16091322/s1. Table S1: Statistical parameters of the distance–decay relationships for metazoan communities. Figure S1: Relative read abundance of major phyla in metazoan communities during wet and dry seasons: (a) relative read abundance of major phyla during the wet season; (b) relative read abundance of major phyla during the dry season. Figure S2. Spatiotemporal variation in metazoan alpha-diversity (ACE Index). Seasonal differences in ACE richness index between the wet and dry seasons within the (a) bay, (b) coastal region, and (c) island region. Spatial differences in ACE richness index among the bay, coastal region, and island region during the (d) wet and (e) dry seasons. Figure S3: Relationships between environmental factors and the relative read abundance of major metazoan phyla.
