# Supplementary material for: Deciphering Metazoan Community Dynamics Using eDNA in a Human-Impacted Gulf Ecosystem: Spatiotemporal Patterns and Environmental Drivers
Source: Animals (Basel). 2026 Apr 26;16(9):1322. doi: 10.3390/ani16091322 (PMC13162636; doi:10.3390/ani16091322)
Supplement: Supplementary file 1 [file animals-16-01322-s001.zip › animals-4252091-supplementary.pdf]

Table S1. Statistical parameters of the distance decay relationships for metazoan communities.

Time	Space	Bray-Curtis			Jaccard		
		Slope	Mantel r	P	Slope	Mantel r	P
Wet	Bay	1.41E-03	0.405	0.001	8.08E-04	0.417	0.001
	Coastal	4.91E-04	0.234	0.017	6.71E-04	0.424	0.001
	Island	-9.29E-04	-0.047	0.61	3.98E-03	0.281	0.122
Dry	Bay	1.55E-03	0.35	0.001	1.33E-03	0.589	0.001
	Coastal	2.92E-04	0.086	0.204	7.47E-04	0.47	0.001
	Island	-2.67E-03	-0.285	0.946	2.91E-03	0.309	0.069

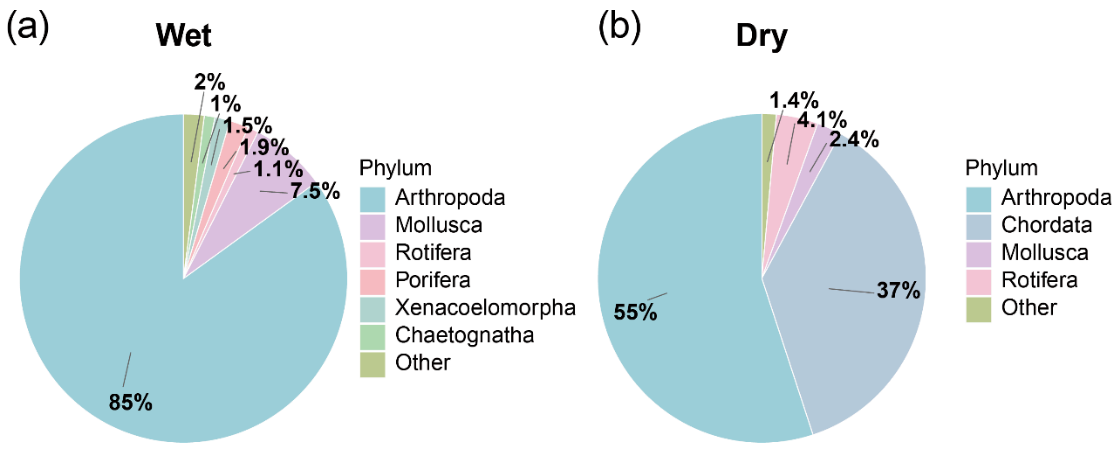

Figure S1. Relative read abundance of major phyla in metazoan communities during wet and dry seasons.

(a) Relative read abundance of major phyla during the wet season. (b) Relative read abundance of major phyla during the dry season. The "Other" category includes phyla with a relative abundance of less than 1%.

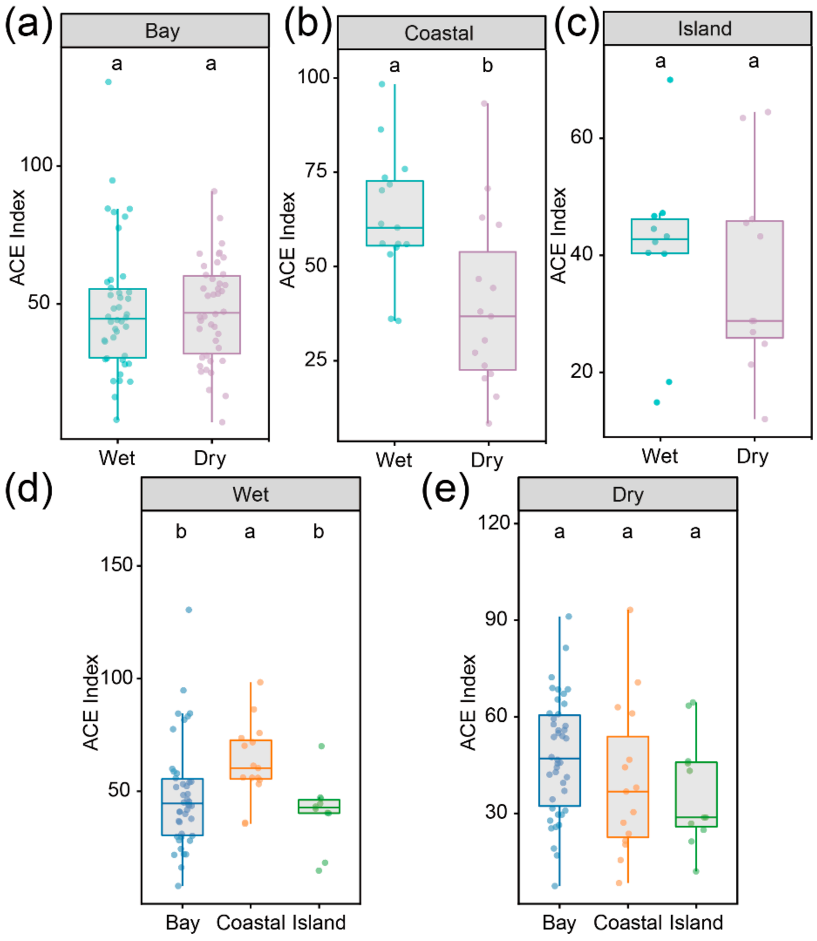

Figure S2. Spatio-temporal variation in metazoan alpha-diversity (ACE Index). Seasonal differences in ACE richness index between the wet and dry seasons within the (a) bay, (b) coastal region, and (c) island region. Spatial differences in ACE richness index among the bay, coastal region, and island region during the (d) wet and (e) dry seasons. Different letters above the boxes indicate statistically significant differences among groups at $p < 0.05$, determined by the Kruskal–Wallis test followed by Dunn's post hoc multiple comparisons test.

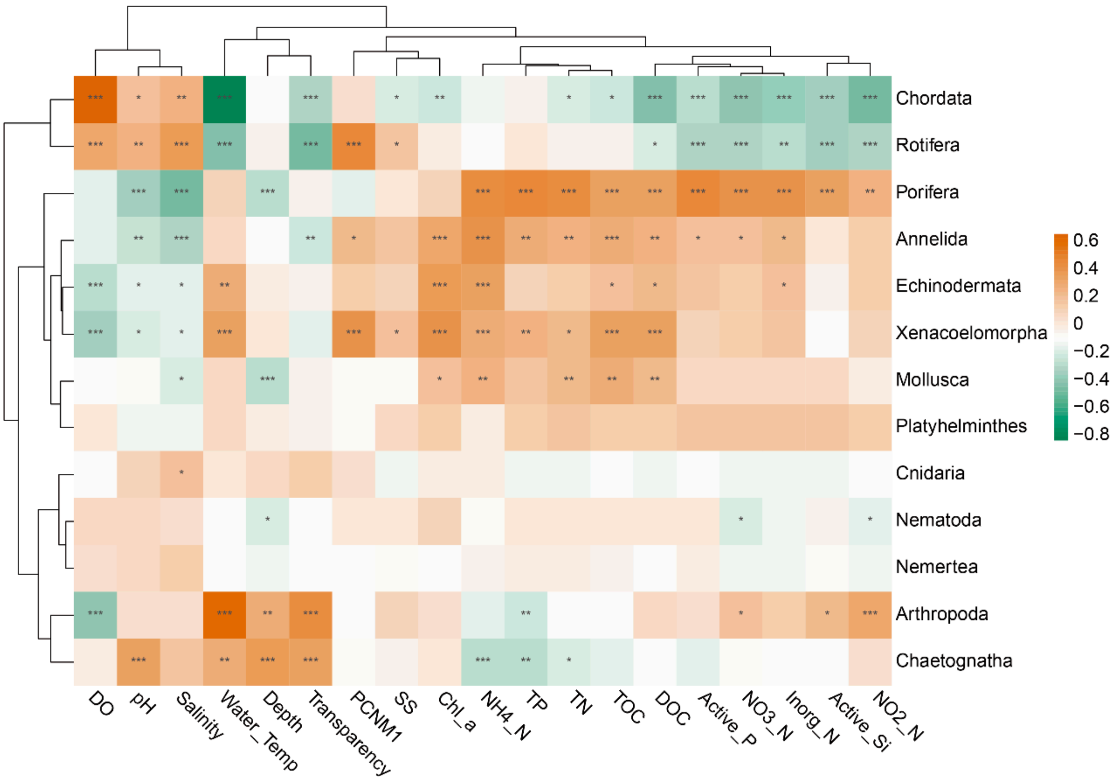

Figure S3. Relationships between environmental factors and the relative abundance of major metazoan phyla.

The Spearman correlation heat map illustrates the associations between environmental variables and the community composition at the phylum level. The color gradient represents the correlation coefficient (ρ), where orange indicates a positive correlation and green indicates a negative correlation. Asterisks denote statistical significance: * $p < 0.05$, ** $p < 0.01$, and *** $p < 0.001$.
